# Temperature and Torque Measurements of Switched Reluctance Actuator with Composite or Laminated Magnetic Cores

**DOI:** 10.3390/s20113065

**Published:** 2020-05-28

**Authors:** Marek Przybylski, Barbara Ślusarek, Paolo Di Barba, Maria Evelina Mognaschi, Sławomir Wiak

**Affiliations:** 1 Łukasiewicz Research Network—Tele and Radio Research Institute, Ratuszowa 11 St., 03-450 Warsaw, Poland; barbara.slusarek@itr.org.pl; 2 Computer and Biomedical Engineering, Department of Electrical, University of Pavia, via Ferrata 5, 27100 Pavia, Italy; paolo.dibarba@unipv.it (P.D.B.); eve.mognaschi@unipv.it (M.E.M.); 3 Institute of Mechatronics and Information Systems, Łódź University of Technology, Stefanowskiego 18/22 St., 90-924 Łódź, Poland; slawomir.wiak@p.lodz.pl

**Keywords:** switched reluctance actuators and motors, thermal measurements, soft magnetic composites, variable speed drives, magnetization characteristics, iron loss measurements

## Abstract

Soft magnetic composite (SMC) materials made of iron powder are more frequently used in construction of electric actuators and motors because of their advantages with respect to Fe–Si electric steel sheets and because they have almost no powder loss. The study deals with measurements of temperature and torque of a low-power rotary switched reluctance actuator, with reference to a commercial actuator and a prototype actuator characterized by stator and rotor cores made of soft magnetic composite materials. Further power loss analysis was also conducted. To assess the actuators, magnetization characteristics and iron loss vs. magnetic flux density at a given frequency were measured according to IEC standards. Results show that the actuator made of soft magnetic composites exhibits higher efficiency and a lower temperature rise of stator and windings in comparison with the commercial actuator.

## 1. Introduction

Nowadays, much effort is devoted to designing new devices that are ecologically friendly in their production and applications. New equipment not only must have better performance than existing equipment, but also has to be efficient. High efficiency of electrical devices causes not only monetary savings for customers, but also causes lower consumption of electric energy, causing lower CO_2_ emissions to the atmosphere during the production of electric energy. Electric propulsion is caused by electric actuators and motors of different constructions, such as, among others, induction, synchronous and brushed DC. Among synchronous actuators and motors, we can distinguish machines with permanent magnets, such as brushless DC motors (BLDC) and permanent magnet synchronous machines (PMSM). Actuators and motors that exploit reluctance force and torque can be divided into synchronous or switched reluctance. Switched reluctance energy converters (linear or rotary) exploit magnetic reluctance force or torque created by attraction of ferromagnetic elements placed in a magnetic field. A switched reluctance motor (SRM) can be treated as a switched reluctance rotary actuator (SRRA). This was first conceived of in the first part of the 19th century; however, real models of such motors have only been manufactured from the mid-1960s. That became possible with the development of switching electronic elements such as thyristors and transistors. Such motors have many advantages such as, among others, the stator and rotor are simply constructed, only the stator has windings and is simple to wind, and there is an absence of permanent magnets. Most losses appear in the stator, which is easy to cool, while the rotor can have a higher temperature than a rotor with permanent magnets, and extremely high speeds are possible. On the other hand, SRM also has disadvantages, such as a pulsed torque with high ripples which can cause acoustic noise. Switched reluctance motors also need an electric converter and they cannot be supplied from an AC line [1].

Electric machine producers are looking for new machine construction methods that will incur a lower cost of materials but have the same operational parameters or—conversely—for design motors with better performances than classical ones [2,3,4]. Modern electrical machines with electronic commutation often use permanent magnets in their construction. Nd–Fe–B and Sm–Co permanent magnets are expensive. That is why increasing efforts have been invested into the development of machines without permanent magnets, especially as a main motor in electric cars [5,6].

Today, washing machines are one of their most important home appliances. According to the Polish Investment and Trade Agency, almost 6.7 million washing machines were produced in Poland in 2018. Each washing machine has at least one motor which is used to rotate a drum. Nowadays, the most common is a universal motor, which is supplied from an AC line. These motors have many advantages, such as very high rotational speed and high starting torque; however, they also have many disadvantages, such as relatively low efficiency: from 30% for the lowest power motors to up to 70% for 2 kW motors. Universal motors have a commutator and brushes which can be inconvenient for users. These motors need commutator maintenance and brush replacement. Working motors could also be a source of electromagnetic interference [7]. For example, in a low-cost washing machine produced by the Amica company, a universal motor with 300 W mechanical power achieved at 14,000 rpm is used. A washing machine drum is driven by a belt gear. The commonly used gear ratio is 14:1.

Nowadays, electric machines with electronic commutation are mainly under development. In recent years, an American company Maytag applied a type H55BMBJL-1820 SRM made by the Emerson Electric Co. in their Neptune MAH 3000 washing machines instead of the commonly used universal motors with a commutator. The Emerson Electric Co. (Ferguson, MO, USA) motor is a 12/8 pole motor with 3 phases. This motor develops high torque at low speed for washing and high speed for spinning. In a washing machine, this motor is supplied and controlled by a microprocessor device. This device is supplied from a 120 V, 60 Hz AC line with a 2.5 A nominal current. The motor control device changes the 120 V AC voltage input to 170 V DC and distributes the current to 3 phases using a classical asymmetric bridge converter. The motor is equipped with a position sensor [8] to ensure adequate supply sequence of phases and current to on and off angles. The motor is connected to the washing machine drum by a belt transmission with a ratio of 14:1. During wash and rinse, the drum rotates at about 50 rpm and during spin at about 800 rpm. This causes rotational rotor speeds of 700 rpm and 11,200 rpm, respectively. This motor’s stator and rotor are composed of electrical steel with 0.65 mm thickness.

New materials developed in recent years are now used in modern electrical equipment. These include the development and application of new soft ferromagnetic materials—mainly magnetic composites. Soft magnetic composite (SMC) is a material that consists of at least two kinds of materials called phases. The first of these is magnetic material and the second is an insulating material. The first material is mainly iron powder particles because of this material’s high maximum permeability (up to µ_rmax_ = 200,000) and it has one of the highest magnetic saturation polarizations (2.15 T). Iron also exhibits low coercivity (4 A/m), causing low hysteresis loss. However, for mass production the most important factor is that it has the lowest price of available powders. The second material is a coating. Coatings can be organic material (e.g., epoxy or polyester resins) or inorganic insulating materials (e.g., phosphates, oxides or sulfates). An insulating material serves two functions: a bonding function to yield high strength and a dielectric function to cause high resistivity of a bulk material. To obtain a compression molding, magnetic composite core powder with coating is pressed in a die. After compression, a green compact is put into a furnace to ensure high strength and partially release stress after compaction. The temperature of curing depends on the kind of dielectric and ranges from approximately 200 to 600 °C [9,10].

The obtained composite core has many advantages in comparison with magnetic circuits made of electrical steel sheets. One of the most important advantages is production of magnetic cores with fewer elements than for those made of laminations. The second is lack of waste; almost 100% of the powder is exploited, whereas during cutting of electrical steel there are always scraps such as grooves or the 4 edges of the stator core. SMC material enables designers of electric machines to design them with complicated shapes that are not possible or very difficult to create with laminations. For higher frequencies of magnetic flux densities, SMCs exhibit lower total loss density, especially because of lower eddy current loss. This is caused by high resistivity of a bulk material. This type of material also has disadvantages such as lower magnetic flux density at the same magnetic field strength and higher hysteresis loss in comparison with electrical steel sheets; and lower thermal conductivity, causing less ability to emit heat from the windings and magnetic core.

The main producers of soft magnetic powders for magnetic composites are Höganäs AB from Sweden, Rio Tinto Metal Powders (formerly QMP) from Canada and GKN Powder Metallurgy from the USA. These companies produce iron powders with a dielectric coating for the production of SMCs. Höganäs produces a few powders called Somaloy, Rio Tinto produces Atomet EM-1 and GKN Powder Metallurgy produces AncorLam powders.

In recent years, many applications of SMC materials have been noted in the electric motor industry. SMC material is mainly seen in transverse or axial flux machines and linear and claw pole motors [11,12,13,14,15,16]. Applications of SMCs in SRMs have also been observed [17,18,19,20].

Much effort has been devoted to the prediction, calculation and measurement of iron loss in SRMs. The first works dealt with, among others, measurement of iron loss [21]. Later works dealt with estimation of core losses. The authors of [22] calculated iron losses in different parts of the magnetic core. They decomposed magnetic flux waveforms into constituent sinusoidal components using Fourier analysis. This method is complicated and a new approach to the calculation of core losses was presented [23]. Here, flux waveforms at each part of the core are expressed in matrix equations of normalized flux pulses of the stator poles. In this method, core losses are divided into hysteresis and eddy current components. Eddy current loss component is calculated from the Steinmetz equation, whereas the hysteresis component is derived from measurements of full and minor hysteresis loops. Further analysis of iron loss was conducted using computer simulations [24]. Here, the authors developed a simulation model and calculated iron loss from the eddy current and hysteresis contributions. The authors of [25] developed a dynamic model of SRM with core losses. In turn, in [26] Ansys 9.0 software was used with the finite element method to build a computer model of SRM and calculate iron losses using the Steinmetz equation. Torrent et al. developed a new method for predicting core losses in SRM [27]. They took into account the type of control used to energize the motor and non-sinusoidal flux waveforms in different parts of the magnetic core. This method takes into account three components of loss: hysteresis, eddy current and excess loss. The finite element method is used for dynamic motor simulations and is associated with the electronic power converter supply algorithm. More recently, the authors of [28] applied a method of fast iron loss calculation to thermal prediction for SRM. In this method, iron loss is calculated by means of a maximum co-energy loop for a defined peak value of current linkage.

SRM fundamental switching frequency of currents is equal to rotational speed per second multiplied by number of rotor poles. For the analyzed SRM with a maximum speed of 10,000 rpm and 8 rotor poles, the base switching frequency of currents is f_bs_ = 1333.3 Hz. An SRM not only has different frequencies of fluxes in parts of the magnetic circuit, but also magnetic fluxes have higher harmonics than a fundamental one. The highest fundamental frequency is observed in some parts of the stator yoke, f_1j_ = 3f_bs_ = 4000 Hz, in other parts of the stator yoke, f_2j_ = f_bs_ = 1333.3 Hz and in the stator poles, f_sp_ = f_bs_ = 1333.3 Hz, whereas in the rotor core f_rc_ = f_bs_/8 = 167 Hz, which is the same in the rotor poles, f_rp_. That is why maximum losses occur in the stator core [21,22,27]. Consequently, application of SMC material in an SRM construction can be attractive as a replacement for electrical steel sheets. SMC materials called Somaloy 700 and Somaloy 700HR were applied in a new construction of an SRM, based on the Emerson Electric Co. motor, for a stator and rotor magnetic core.

The main aim of this research was to compare the performance of motors with thermal measurements and analyze power loss of the modified Emerson Electric Co. motor and the motor developed with SMC material. Emerson Electric motor windings were modified so that both motors were able to be supplied from a controller developed by the Poznan University of Technology and supplied from Poland’s electricity system with 230 V AC, 50 Hz [29,30].

## 2. Materials and Methods

An SRM with a magnetic circuit made of SMC material was designed and built. The motor was designed for the Polish electrical supply system—230 V and 50 Hz. For the sake of comparing the two motors, a configuration of coils in windings in the Emerson Electric motor were changed. The change enabled an increase of the supply voltage from 120 V to 230 V. Originally, the Emerson Electric motor had two parallel connected coils that were modified and connected in series with the next two coils, also originally connected in parallel. This way, the modified motor and the soft magnetic composite motor had all phase coils connected in series.

### 2.1. Soft Magnetic Composites for SRM

Components of the developed motor were made in the Tele and Radio Research Institute in Warsaw. They were made of iron powders called Somaloy 700 and Somaloy 700HR made by Höganäs AB. The magnetic parts consisted of soft magnetic iron powder with a dielectric that surrounds particles of powder with an average size of about 250–300 µm [31]. An SRM with a composite stator made of Somaloy 700 iron powder and with a composite rotor made of Somaloy 700HR iron powder were developed. The stator and a rotor had dimensions larger than our technical capabilities and were composed from smaller parts. Parts of the stator’s motor were compacted under pressure at 700 MPa, whereas parts of rotor’s motor from the mentioned powders were compacted under 800 MPa. All parts were cured to ensure high mechanical strength and thermal annealing at 530 °C in an air atmosphere for 1 h. The desired shapes of the motor parts were machined or cut using electrical discharge machining. The stator and the rotor magnetic cores were glued together from the obtained parts.

### 2.2. SRM Motor Design

**Figure 1:**
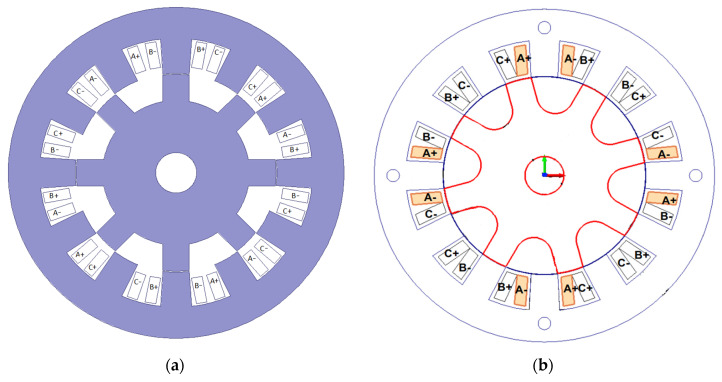
(**a**) Modified Emerson Electric motor structure; (**b**) developed motor with soft magnetic composite (SMC) rotor and stator [32].

Figure 1a shows the structure of the SRM made by the Emerson Electric Co. (Ferguson, MO, USA)., whereas Figure 1b presents the developed one. Both stators have the same dimensions; small differences are found in the motor rotors. The developed motor has 0.25 mm air–gap thickness, whereas the Emerson Electric motor has 0.40 mm air–gap thickness. During the design process, the number of turns in the coils were decreased in the prototype motor to ensure similar electromagnetic behavior of motors with a smaller air–gap thickness in the prototype.

**Figure 2:**
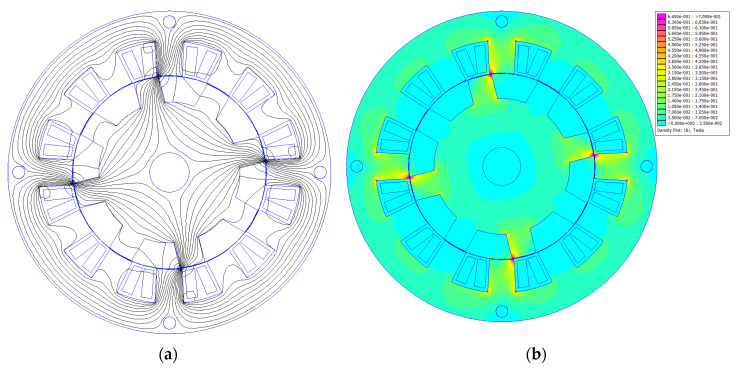
(**a**) Magnetic flux lines for the motor from Figure 1a, I = 1.25 A; (**b**) magnetic flux density for the motor from Figure 1a, I = 1.25 A, B_max_ = 7·10^−1^ T [34].

Figure 2 shows an example of magnetic flux lines and magnetic flux distribution for the modified Emerson Electric motor. Simulations were prepared in FEMM 4.2 [33] software for current in phase B, I = 1.25 A and an angle between stator and rotor poles equal to 14°. This is the angle for maximum torque in a motor T = 0.47 Nm at I = 1.25 A. The simulation region was discretized into approximately 100,000 elements with 50,000 nodes. FEMM 4.2 software uses a conjugate gradient solver with the nonlinear Newton–Raphson method; the simulation in Figure 2 took 6 iterations. Numerical calculations were conducted in 2D, assuming an axial length of the magnetic core equal to 47.3 mm. Because of the quite long axial length of the magnetic core and short end windings, during 2D calculations the end windings were neglected.

As can be seen from Figure 2, the motor has 4 magnetic poles and the magnetic flux density in the teeth edges achieves about 0.7 Tesla.

Table 1 shows the main parameters of the modified Emerson Electric Co., (Ferguson, MO, USA) motor and the prototype.

### 2.3. Methodology of SRM Measurements

Electric motor thermal behavior can be modeled by means of FEM codes [35], known thermal material parameters and sources of heat, which can be very difficult to obtain for SRM motors. The last test of an electric motor design should be thermal measurements of a prototype. Measurements of temperature in a motor can be made directly using thermometers or indirectly; for example, winding temperature measurements can be determined by resistance before and after a thermal test. For thermal characterization of both motors, the temperature of a stator and winding was measured. Temperature was measured using a 2-channel digital thermometer made by CHY, model 502A. A type K thermocouple was placed in an end winding, whereas a type T thermocouple was placed on an outer part of a stator. The thermocouples were produced by Termoaparatura Wroclaw. The temperature of the winding after a thermal test was also calculated from resistance measurements before and after the thermal test. Resistances were measured using the Fluke 115 multimeter.

**Figure 3:**
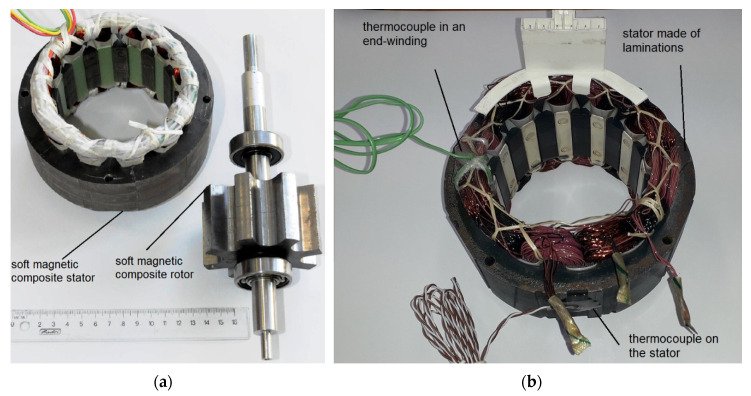
(**a**) Stator and rotor of the developed motor [36]; (**b**) stator of the modified Emerson Electric motor with thermocouples in an end winding and on the stator.

Figure 3a shows a rotor and stator photo of the developed motor. Somaloy 700 was used to manufacture the stator magnetic core, whereas Somaloy 700 HR was used for the rotor magnetic core. The prototype motor used parts from a commercial motor, such as a shaft and bearings. Figure 3b shows a stator of the modified Emerson Electric motor with thermocouples in an end-winding and on outer part of the stator. Both thermocouples were affixed using a glue with high thermal conductivity.

**Figure 4:**
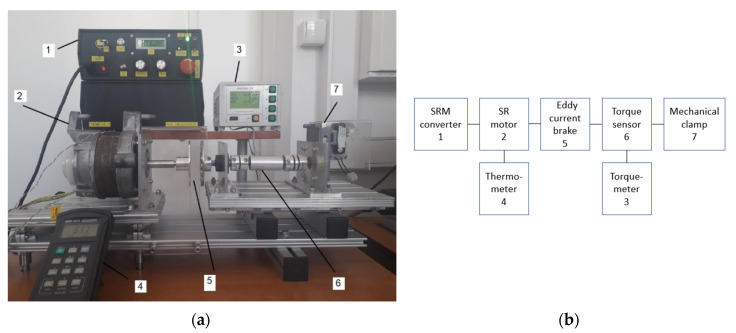
(**a**) Measuring stand with the SMC prototype motor; (**b**) schematic diagram of the measuring stand.

Load and thermal tests of the SRMs were performed using a measuring stand shown in Figure 4. The drive was loaded using an eddy current brake with permanent magnets. The motor was supplied from a 3-phase asymmetric bridge controller designed and developed by the Poznan University of Technology [29,30,37]. Torque was measured with a MW2006-3S torque meter with a MT-3Nm sensor produced by Roman Pomianowski Pracownia Elektroniki (Poznań, Poland). Rotational speed was measured by a meter in the motor controller. Input parameters of the drive, such as AC voltage, current and power were measured by an IT9121 digital power meter produced by ITECH (New Taipei, Tajwan). Input DC voltage and DC current of the asymmetric bridge converter were measured using a Fluke 115 multimeter. Motor phase voltages were measured and recorded using a Tektronix TDS 210 oscilloscope with a TT-HV 250 high voltage probe produced by Testec, (Frankfurt, Germany). Motor phase currents were measured and recorded with a Tektronix TDS 210 oscilloscope with use of the Tektronix A622 AC/DC current probe. Motor input current RMS value was measured with the use of a Fluke 115 multimeter.

Measurements of parameters of magnetic materials were conducted using a AMH-20K-HS model hysteresis graph made by Laboratorio Elettrofisico Engineering (Nerviano, Italy). Measurements were carried out on a ring sample in steel sheets from the Emerson Electric motor with an outer diameter of 60 mm, inner diameter of 40 mm and composed of 13 sheets of 0.65 mm thickness each; whereas measurements were carried out for Somaloy 700 and Somaloy 700HR on ring samples with an outer diameter of 75 mm, inner diameter of 55 mm and height of 10 mm. Each sample was wound with two windings for magnetic flux density, magnetic field strength and iron loss measurements, chosen according to IEC standards. Measurement accuracy of magnetic flux density and magnetic field strength was equal 2%. Measurement accuracy of total iron loss density was equal to 3%.

Figure 4 shows a measured motor (2), eddy current brake (5) with torque sensor (6) with a torque meter (3), SRM controller (1) and digital thermometer (4). One end of the torque meter shaft was held by a mechanical clamp (7) and could not rotate; the other was connected to a disc with permanent magnets. This disc is a part of the eddy current brake. A bearing node was used between the eddy current brake and the torque sensor, causing higher stiffness of the mechanical stand.

## 3. Results

In the first part of this research study, magnetization and iron loss characteristics were measured. In the second part of the research study, we modified an Emerson Electric motor and a prototype made with a soft magnetic composite core was measured. Measurements of drive performance such as torque vs. rotational speed, mechanical power vs. rotational speed and thermal behavior of motors loaded with the same speed and torque were taken.

### 3.1. Measurement of Magnetization and Iron Loss Characteristics

DC characteristics of magnetic flux density as a function of magnetic field strength B = f(H) were measured. Then, iron loss characteristics vs. magnetic flux density and its frequency B = f(H) were taken according to the IEC standard 60,404–4 on a ring sample, whereas iron loss characteristics were taken according to the IEC standard 60,404–6, also on a ring sample.

Because the largest iron loss was observed in the stator poles and in parts of the core with frequency f_2j_, iron loss was measured for this frequency. Although the highest frequency was observed in parts of the stator core and is equal to 3 times the base switching frequency, it did not cause the highest contribution to iron loss. This was connected to a low amplitude of changes in magnetic flux.

A thermal test and analysis and main drive performance analysis were conducted for rotational speed *n* = 4400 rpm to determine if iron loss existed in a sample cut from Emerson Electric motor; in turn, for a sample made of Somaloy 700 was measured at 600 Hz for the stator core and stator poles and at 50 Hz for rotor poles and the rotor core.

**Figure 5:**
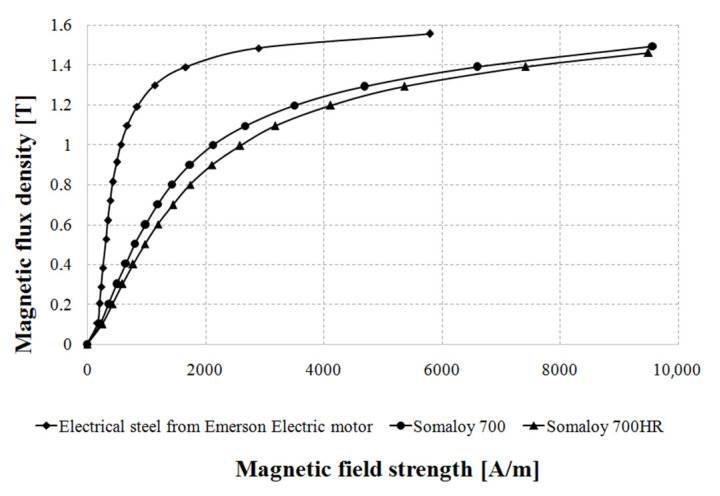
DC magnetization curves for Emerson Electric steel, Somaloy 700 and Somaloy 700HR.

Figure 5 shows magnetization curves for electrical steel cut from the Emerson Electric motor, Somaloy 700 and Somaloy 700HR.

Figure 5 shows that, of all soft magnetic materials, electrical steel from the Emerson Electric motor exhibits the highest magnetic flux density for the same magnetic field strength. Maximum relative magnetic permeability for Emerson Electric steel µ_r_ = B/(µ_0_H) = 1485 and is the highest of all materials; for Somaloy 700, µ = 497 and for Somaloy 700HR, µ = 412. Because permeability for Emerson Electric steel is the highest, for the prototype motor the air–gap was changed from 0.4 mm to 0.25 mm.

**Figure 6:**
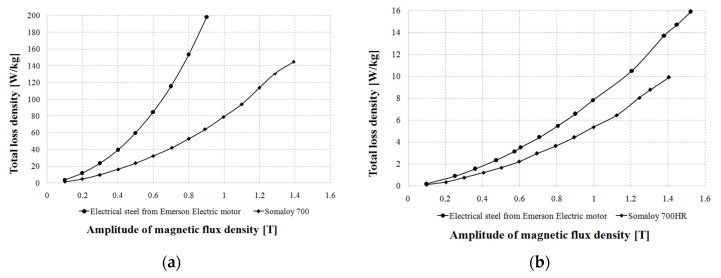
(**a**) Graphs of iron loss density vs. magnetic flux density for electrical steel from the Emerson Electric motor and for Somaloy 700 materials measured at 600 Hz; (**b**) graphs of iron loss density vs. magnetic flux density for electrical steel from the Emerson Electric motor and for Somaloy 700HR materials measured at 50 Hz.

Figure 6a shows curves of iron loss density as a function of magnetic flux density for electrical steel from the Emerson Electric motor and for Somaloy 700 materials measured at 600 Hz. In turn, Figure 6b shows curves of iron loss density as a function of magnetic flux density for electrical steel from the Emerson Electric motor and for Somaloy 700HR materials measured at 50 Hz.

As can be seen from Figure 6a, iron loss density is higher in steel sheets from the Emerson Electric motor in comparison with Somaloy 700 material at the same magnetic flux density at the frequency 600 Hz. For example, for 0.8 Tesla electrical steel exhibits almost 3 times higher total loss density in comparison with Somaloy 700 material. Comparison of iron loss density for the same magnetic flux density in Figure 6b for 50 Hz shows that Emerson Electric motor sheets have 1.5 times higher loss than Somaloy 700HR.

### 3.2. Measurements of Drive Performance

**Figure 7:**
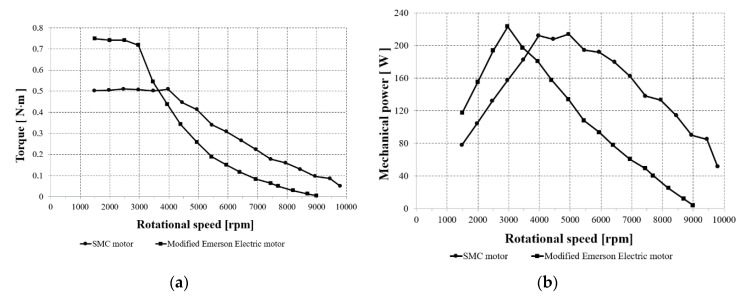
(**a**) Torque vs. rotational speed for the prototype and modified Emerson Electric motors; (**b**) mechanical power vs. rotational speed for the prototype and modified Emerson Electric motors, U_DC_ = 310 V.

Two drives with SRMs were measured. Figure 7a shows curves of the maximum torque as a function of rotational speed. In turn, Figure 7b shows mechanical power vs. rotational speed.

As can be seen from Figure 7a, the modified Emerson Electric motor develops higher maximum torque at low speeds up to 3800 rpm, whereas the prototype develops higher maximum torque for speeds higher than 3800 rpm. The modified Emerson Electric motor could be applied to applications working at low speeds, whereas the prototype could be used for high speeds. Figure 7b shows that the modified Emerson Electric motor develops a higher mechanical power than the SMC motor up to speeds of 3800 rpm, whereas for higher speed the SMC motor is better, and it can develop higher power.

Table 2 shows the results of current measurements supplying motor windings for the two aforementioned motors. Measurements were taken at the rotational speed *n* = 4400 rpm and torque T = 0.35 Nm. Both motors were supplied from U_DC_ = 310 V. The modified Emerson Electric motor has smaller value supply currents because it has higher phase inductance and resistance.

**Figure 8:**
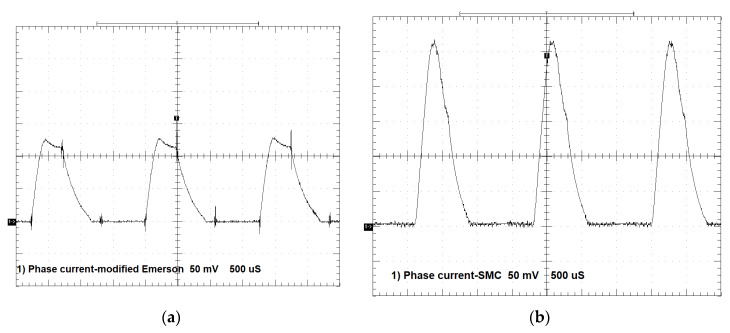
(**a**) Phase A current vs. time for the modified Emerson Electric motor, T = 0.35 Nm, *n* = 4400 rpm, 1 div = 0.5 A, 1 div = 500 µs; (**b**) phase A current vs. time for the prototype motor, T = 0.35 Nm, *n* = 4400 rpm, 1 div = 0.5 A, 1 div = 500 µs.

Figure 8a shows phase current vs. time for the modified Emerson Electric motor, whereas Figure 8b shows phase current vs. time for the prototype motor measured for the same loading torque T = 0.35 Nm and rotational speed *n* = 4400 rpm. For both motors U_DC_ = 310 V.

Figure 8a shows that, for the modified Emerson Electric motor, peak current I_p_ = 1.3 A, whereas for the SMC motor, I_p_ = 2.75 A. As can be seen, the SMC motor is supplied with higher current in comparison with the modified Emerson Electric motor. It is connected to higher phase resistance and inductance with respect to the modified Emerson Electric motor. The frequency of both currents is equal to approximately 580 Hz. The dwell angle for the both motors is equal Θ_D_ = 12.9°.

**Figure 9:**
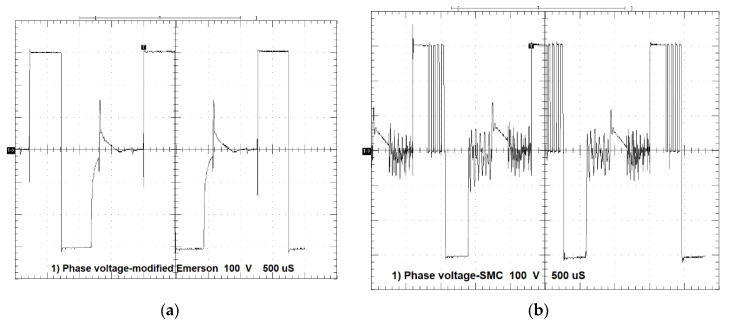
(**a**) Phase A voltage vs. time for the modified Emerson Electric motor, T = 0.35 Nm, *n* = 4400 rpm, 1 div = 100 V, 1 div = 500 µs; (**b**) phase A voltage vs. time for the prototype motor, T = 0.35 Nm, *n* = 4400 rpm, 1 div = 100 V, 1 div = 500 µs.

Figure 9a shows phase voltage for the modified Emerson Electric motor, whereas Figure 9b shows phase voltage for the SMC motor.

As can be seen from Figure 9, phase voltages of the two motors are similar, both in terms of the maximum applied voltages U_DC_ = 310 V for conduction time and the same voltage U_DC_ = −310 V for non-conduction time. In the case of the prototype motor, we can see that the chopping of voltage keeps the current constant, whereas for the modified Emerson Electric motor there is constant conduction.

Table 3 shows the performance of the two drives at the same torque T = 0.35 Nm and rotational speed *n* = 4400 rpm. Both drives were supplied from an AC line: U = 230 V, f = 50 Hz.

For the same mechanical power, the SMC motor has 8% higher efficiency than the modified Emerson Electric motor; however, the total harmonic distortion of current is 8% higher.

**Figure 10:**
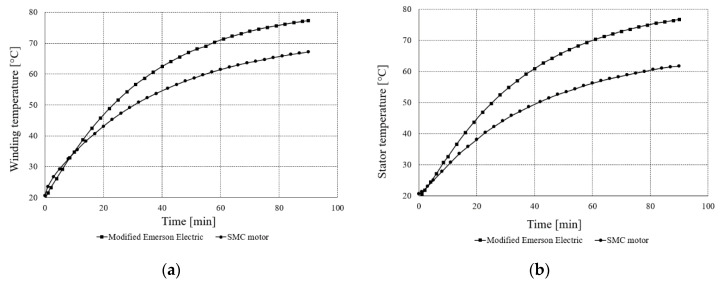
(**a**) Temperature of windings for the modified Emerson Electric motor and SMC motor; (**b**) temperature of stators for modified Emerson Electric motor and SMC motor, T = 0.35 Nm, *n* = 4400 rpm.

Figure 10a shows, in turn, winding temperature measurements of the two motors, whereas Figure 10b shows measurements of stator temperature at the torque T = 0.35 Nm and *n* = 4400 rpm.

As can be seen from Figure 10a, the temperature of the end winding of the modified Emerson Electric motor was higher than that of the SMC motor during the thermal test after 8 min. After 90 min at the test temperature, the winding of the modified Emerson Electric motor was higher by about 10 °C. The modified Emerson Electric motor winding reached a temperature of about 80 °C, whereas the SMC motor reached about 70 °C. When checked after the thermal test, the average temperature of three phases of the modified Emerson Electric winding reached a temperature of 80.1 °C, whereas the temperature of the SMC motor was 65.2 °C. Figure 10b shows the stator temperature of the two motors. Stator temperature of the modified Emerson Electric motor was higher than the SMC motor for the whole measurement time. After 90 min of thermal testing, the temperature of the modified Emerson Electric stator was 15 °C higher than the SMC motor. The modified Emerson Electric motor reached a temperature of 80 °C, whereas the SMC motor reached 65 °C.

After measurement, a rough determination of iron power loss was conducted for T = 0.35 Nm and *n* = 4400 rpm; this is shown in Table 4. Neglecting power loss in the converter, windage and friction loss for the two motors’ iron loss can be roughly estimated. Copper loss was calculated from the RMS current in the second power phase multiplied by the resistance of the winding and number of phases [22].

After calculation of losses in the two motors, it can be seen that iron loss in the modified Emerson Electric motor is 60% higher than in the SMC motor. In turn, copper losses are approximately 40% higher for the SMC motor in comparison with the modified Emerson Electric motor. This is connected to a higher pulsed current because of the lower inductance and resistance of the SMC motor.

## 4. Discussion

The results show that the prototype motor made of soft magnetic composites exhibits better behavior than the modified Emerson Electric motor with respect to higher efficiency and lower iron loss. The obtained efficiency of 71% for the SMC motor is 8% higher than that of the modified Emerson Electric motor for *n* = 4400 rpm and T = 0.35 Nm. Comparison of iron loss in the motors showed that the modified Emerson Electric motor has 60% higher iron loss than the SMC motor for the same conditions. This is connected to higher iron loss density in the Emerson Electric motor steel in comparison with Somaloy 700 and Somaloy 700HR materials for the same magnetic flux density and frequency. Copper loss of the SMC motor is higher than in the modified Emerson Electric motor by about 40%. The thermal behavior of motors showed that the SMC motor exhibits lower temperature for the stator and end windings in comparison with the modified Emerson Electric motor by approximately 10 °C for windings and 15 °C for the stator at a steady state.

As presented, the thermal loadings of motors are low. This research will be useful to help elaborate a new, optimized motor with a lower-weight magnetic core made of composite materials. This study also presents measurements that show that soft magnetic composite material has lower iron loss at the same frequency and magnetic flux density, although it has lower permeability.

In future works, loss and thermal analysis of drives will be conducted, taking into account iron loss vs. magnetic flux densities; frequency characteristics, windage and friction loss; and losses in the converter.

## Figures and Tables

**Table 1:** Parameters of modified Emerson Electric motor and prototype made of soft magnetic composites (SMCs).

Parameter	Modified Emerson Electric Motor	Prototype Made of SMCs
DC supply voltage	310 V	310 V
Max. rotational speed	9000 rpm	9800 rpm
External stator diameter	139.5	139.5
External rotor diameter	83 mm	83 mm
Axial length of magnetic core	47.3 mm	46.6 mm
Air gap length	0.4 mm	0.25 mm
Number of phases	3	3
Number of stator poles	12	12
Number of rotor poles	8	8
Material of stator core	Steel lamination	Somaloy 700
Material of rotor core	Steel lamination	Somaloy 700HR
Phase resistance	8.4 ± 0.3 Ω	5.7 ± 0.3 Ω
Coil turns	172	134
Wire diameter	0.56 mm	0.60 mm
Phase inductance—aligned	242 mH	139 mH
Phase inductance—unaligned	36 mH	24 mH
Stator pole angle	15.5°	15.5°
Rotor pole angle	16°	16°

**Table 2:** Phase supply currents for modified Emerson Electric and SMC motors, for T = 0.35 Nm, *n* = 4400 rpm.

Type of Motor	DC Phase Voltage [V]	RMS Current [A]	Average Current [A]	Peak Current [A]
Modified Emerson Electric	310 ± 4	0.67 ± 0.02	0.43 ± 0.01	1.30 ± 0.09
SMC	310 ± 4	0.96 ± 0.02	0.63 ± 0.01	2.75 ± 0.14

**Table 3:** Drive performance with modified Emerson Electric and SMC motors, for T = 0.35 Nm, *n* = 4400 rpm.

Type of Motor	AC Supply Voltage [V]	AC I_RMS_ [A]	Input Power [W]	Output Power [W]	Efficiency [%]	Current THD [%]
Modified Emerson Electric	230 ± 1	1.83 ± 0.01	256 ± 1	161 ± 8	63 ± 4	126
SMC	230 ± 1	1.71 ± 0.01	229 ± 1	161 ± 8	71 ± 4	134

**Table 4:** Power loss for modified Emerson Electric and SMC motors for T = 0.35 Nm, *n* = 4400 rpm.

Type of Motor	Input Power [W]	Output Power [W]	Copper Loss [W]	Iron Loss [W]
Modified Emerson Electric	256 ± 1	161 ± 8	11.3 ± 0.8	83.7 ± 8.2
SMC	229 ± 1	161 ± 8	15.8 ± 1.1	52.2 ± 8.2
